# Supplementary material for: Cytotoxicity Profiles and Neuroprotective Properties of the Novel Ifenprodil Analogues as Sigma Ligands
Source: Molecules. 2023 Apr 13;28(8):3431. doi: 10.3390/molecules28083431 (PMC10146949; doi:10.3390/molecules28083431)

Supplementary Material

Cytotoxicity Profiles and Neuroprotective Properties of the Novel Ifenprodil Analogues as Sigma Ligands

Daniele Zampieri [1,*], Antonella Calabretti [1], Maurizio Romano [2], Sara Fortuna [3], Simona Collina [4], Emanuele Amata [5], Maria Dichiara [5], Agostino Marrazzo [5] and Maria Grazia Mamolo [1]

[1] *Department of Chemical and Pharmaceutical Sciences, University of Trieste, Via Giorgieri 1, 34127 Trieste, Italy*

[2] *Department of Life Sciences, University of Trieste, Via Valerio 28, 34127 Trieste, Italy*

[3] *Italian Institute of Technology (IIT), Via E. Melen 83, 16152 Genova, Italy*

[4] *Department of Drug Sciences, Medicinal Chemistry and Pharmaceutical Technology Section, University of Pavia, Viale Taramelli 6 and 12, 27100 Pavia, Italy*

[5] *Department of Drug and Health Sciences, University of Catania, Viale Doria 6, 95125 Catania, Italy*

* Corresponding author; *email address:* dzampieri@units.it *(D. Zampieri)*

Table of contents

-Chemistry:

-^{1}H-NMR and ^{13}C-NMR Spectra for compounds **5a–o**

-Biology:

-Cytotoxicity curves for compounds **5d**, **5e**, **5h,** **5i**, **5k** and **5o**

^{1}H-NMR and ^{13}C-NMR spectra for compound **5a** ($CDCl_3$)

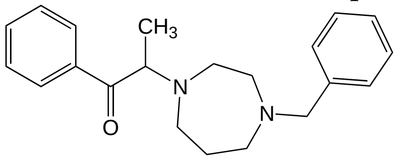

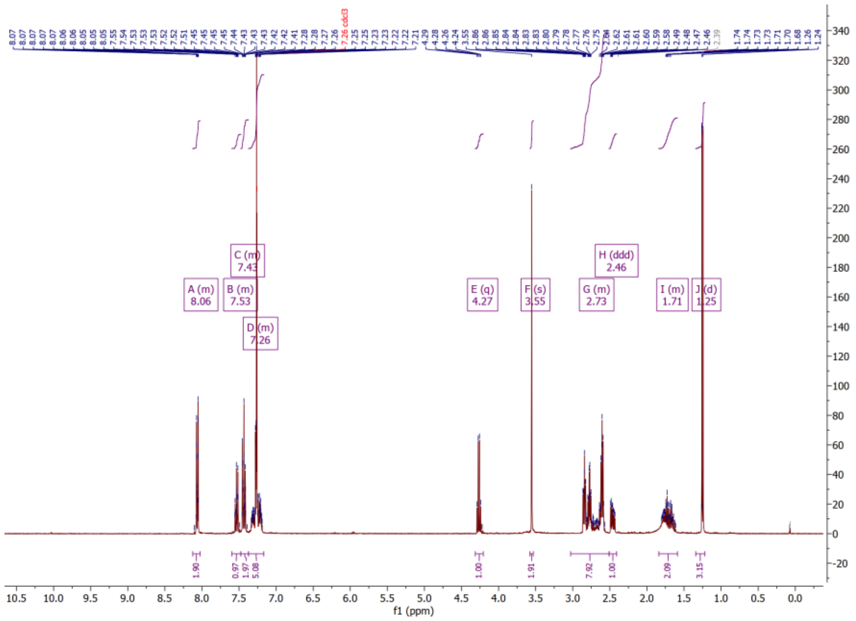

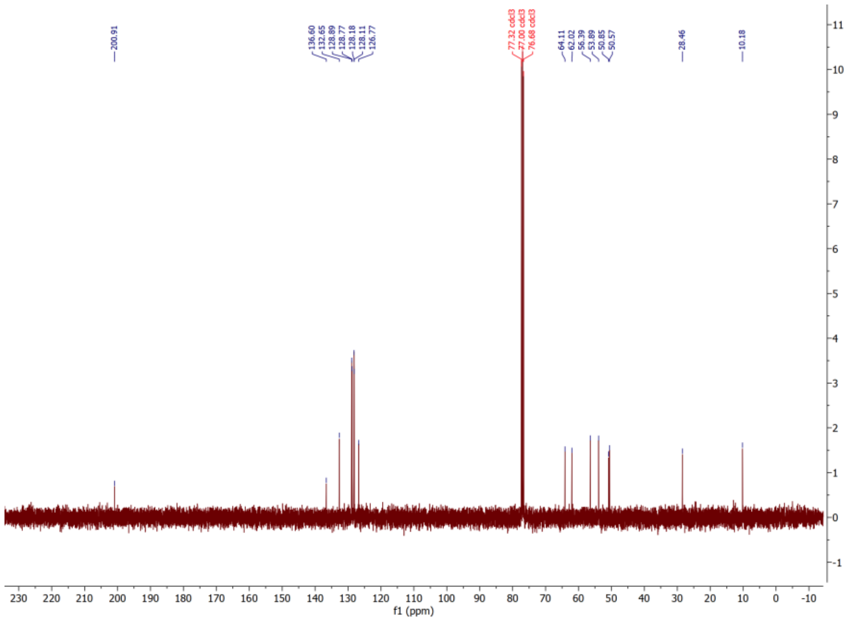

^{1}H-NMR and ^{13}C-NMR spectra for compound **5b** ($CDCl_3$)

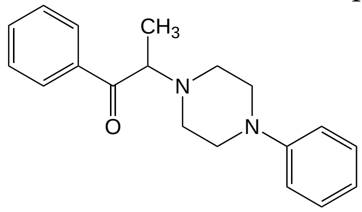

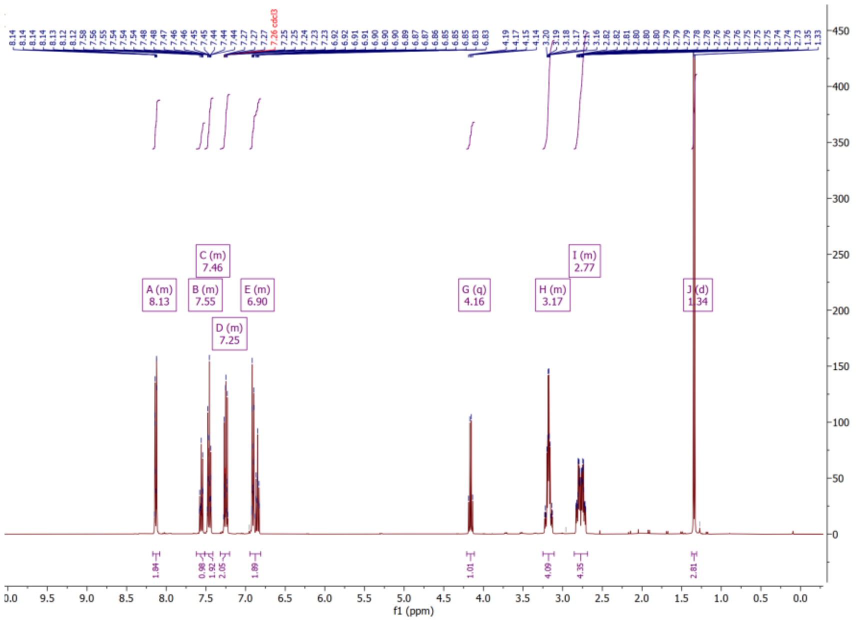

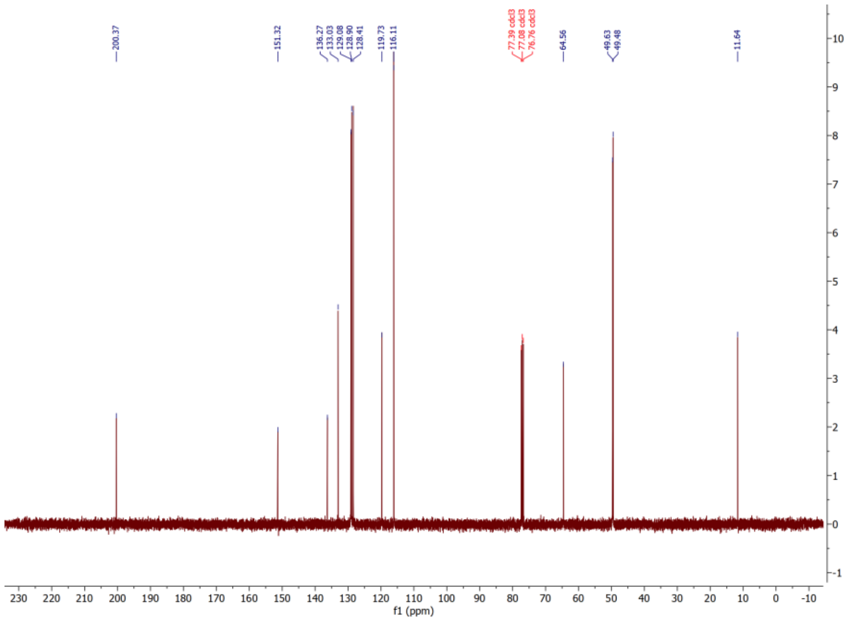

^{1}H-NMR and ^{13}C-NMR spectra for compound **5c** ($CDCl_3$)

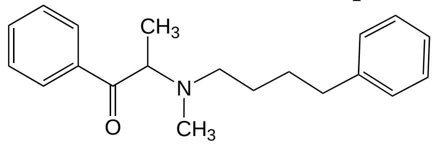

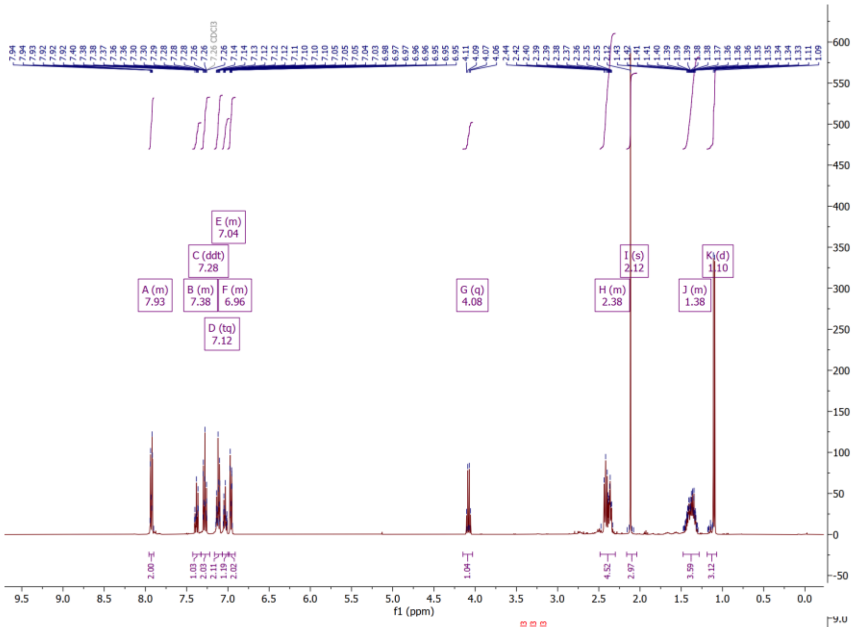

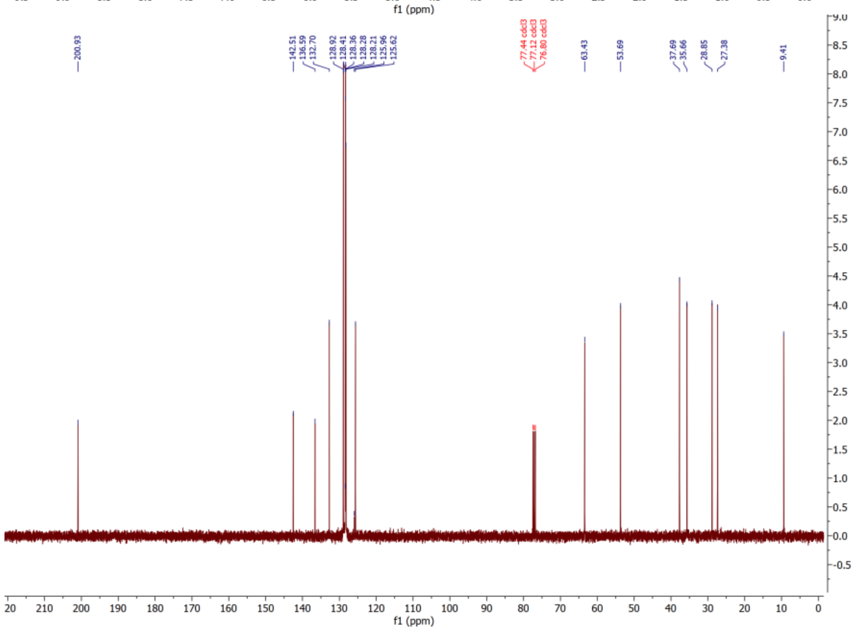

^{1}H-NMR and ^{13}C-NMR spectra for compound **5d** ($CDCl_3$)

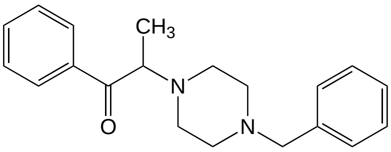

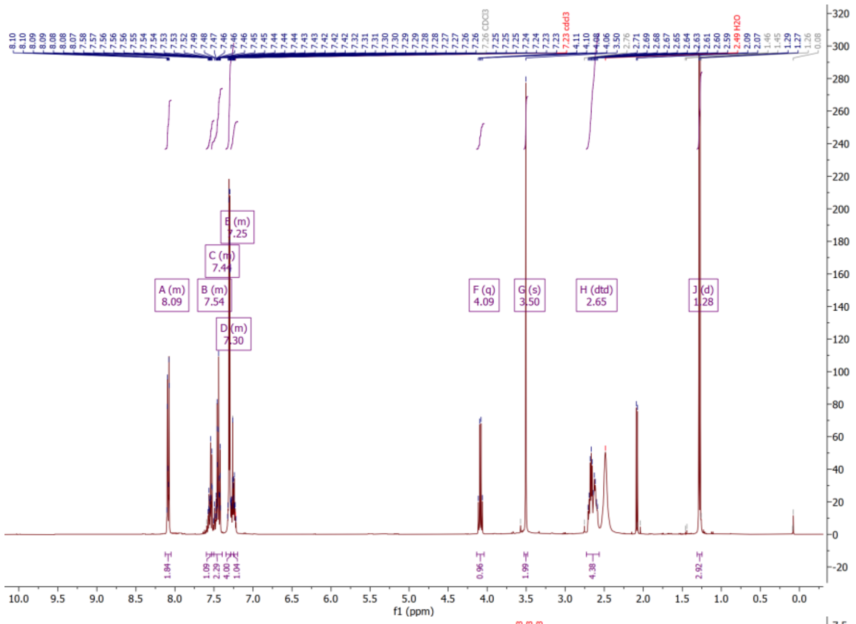

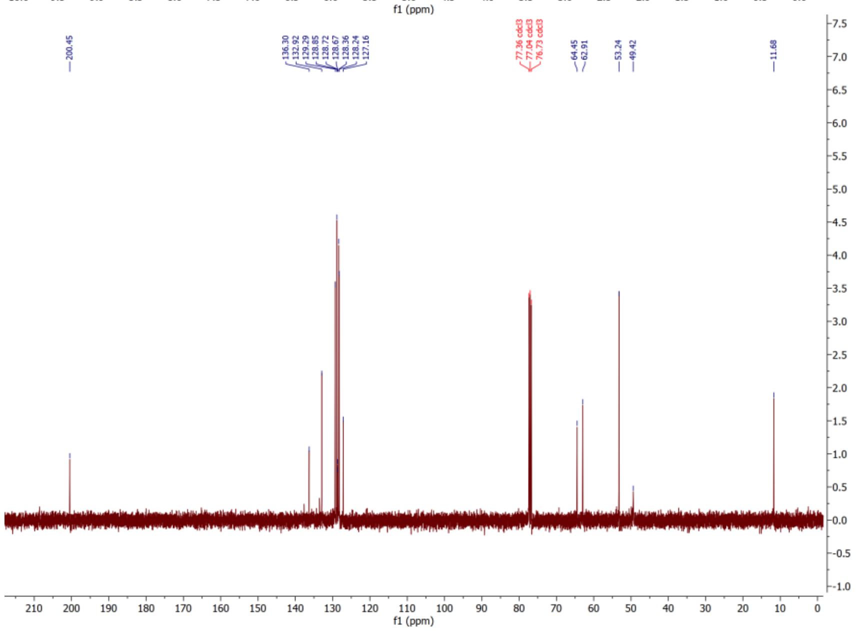

^{1}H-NMR and ^{13}C-NMR spectra for compound **5e** ($CDCl_3$)

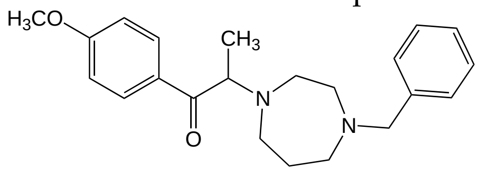

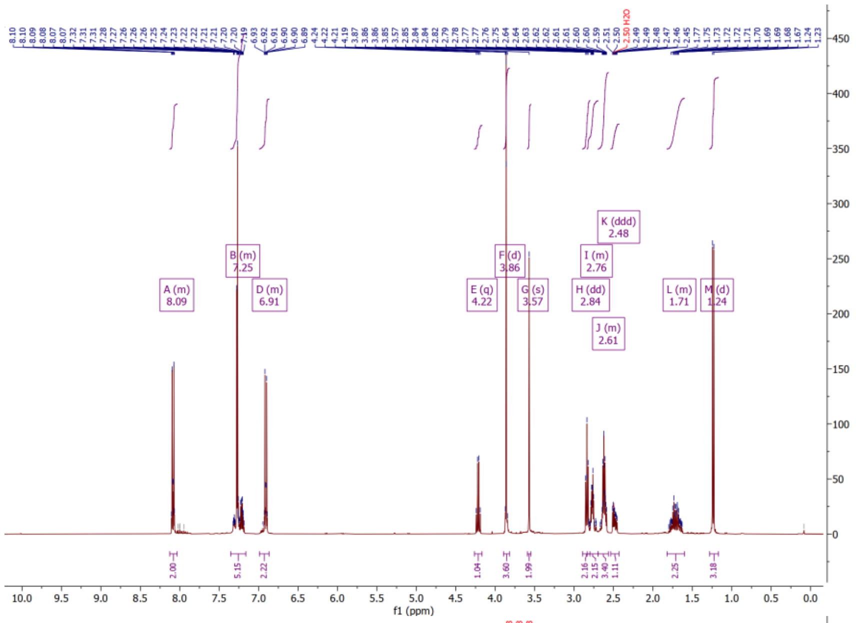

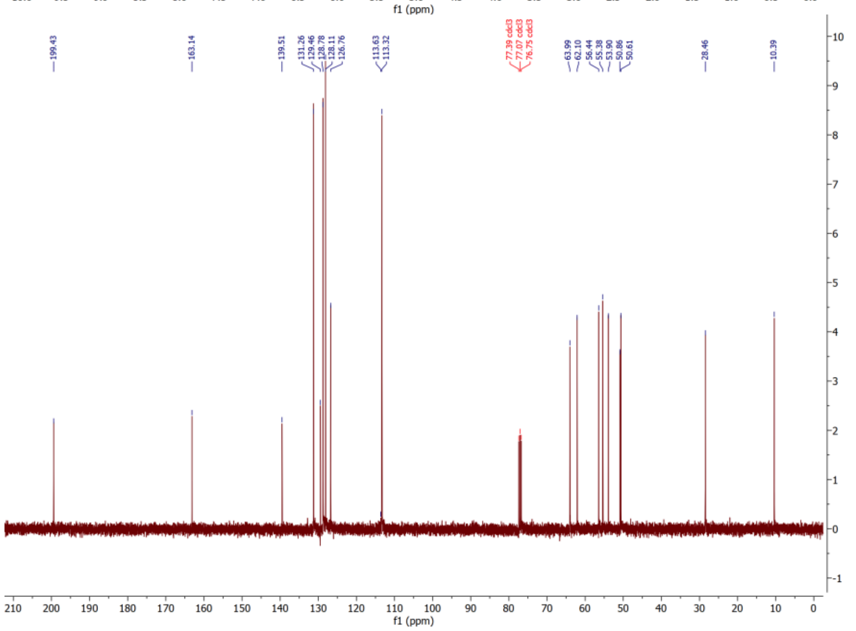

^{1}H-NMR and ^{13}C-NMR spectra for compound **5f** ($CDCl_3$)

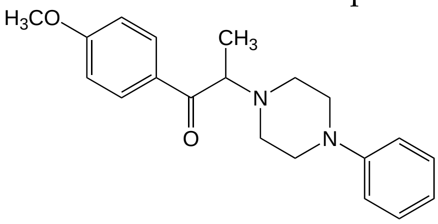

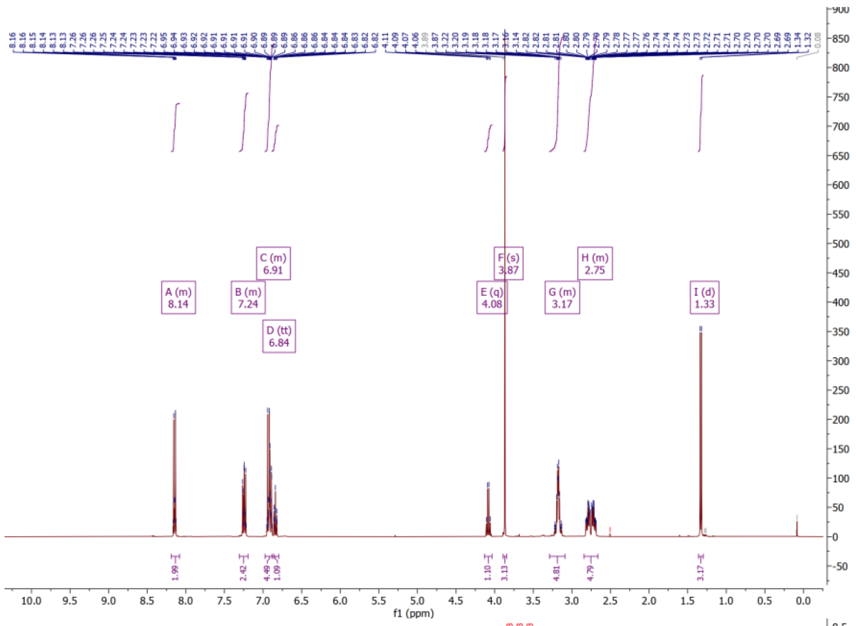

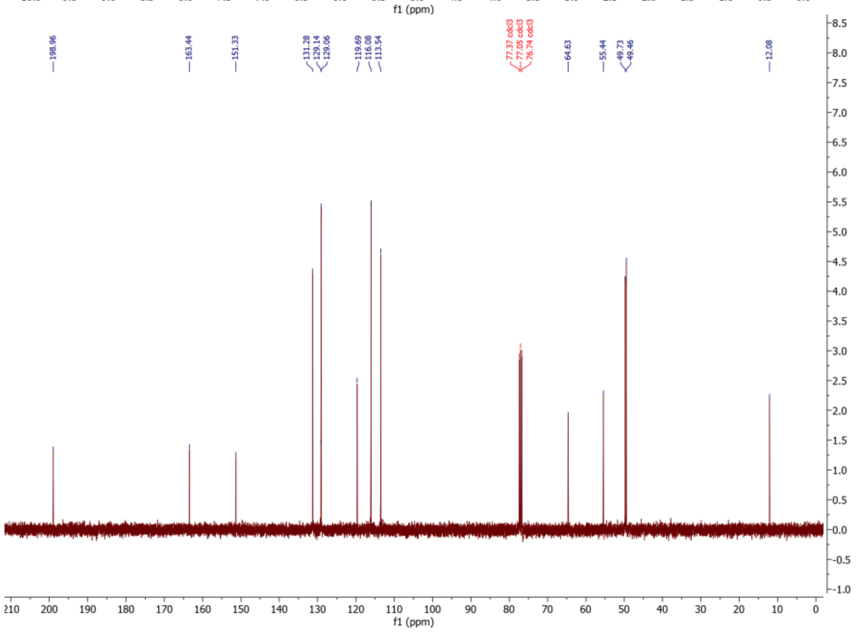

^{1}H-NMR and ^{13}C-NMR spectra for compound **5g** ($CDCl_3$)

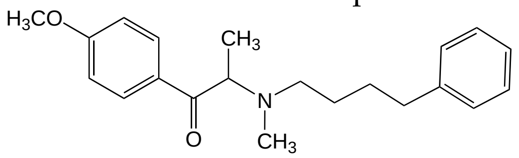

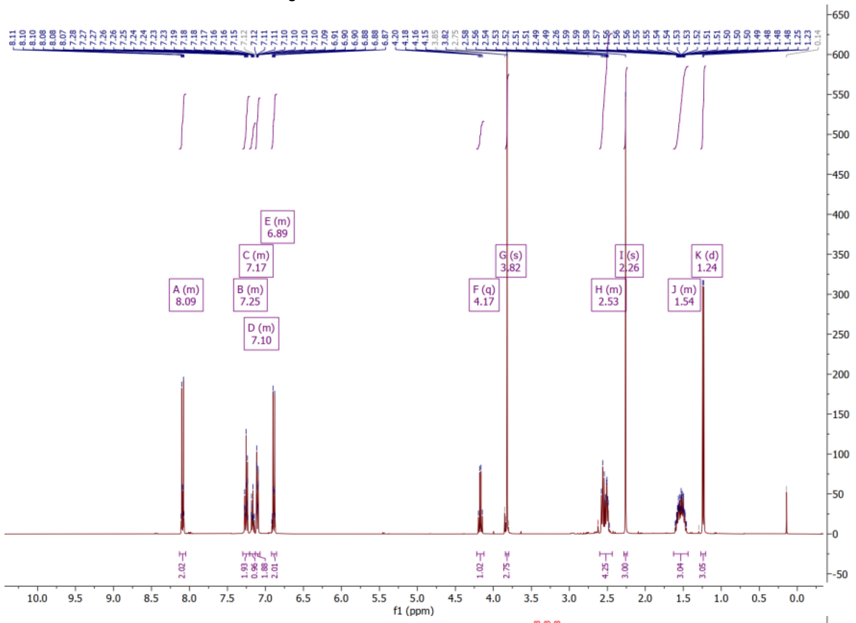

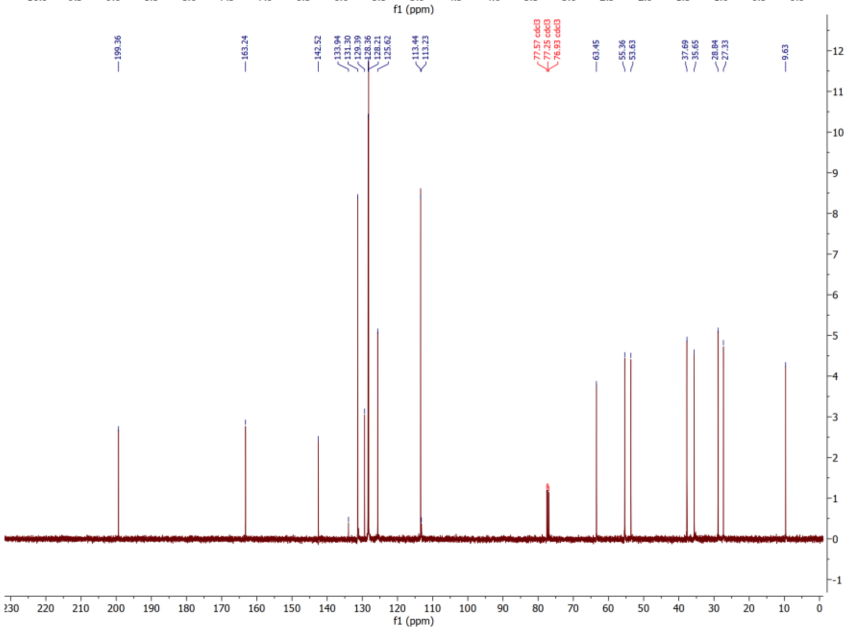

^{1}H-NMR and ^{13}C-NMR spectra for compound **5h** ($CDCl_3$)

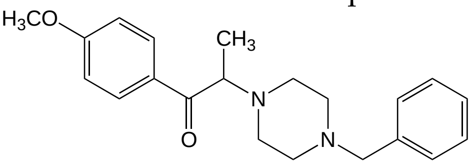

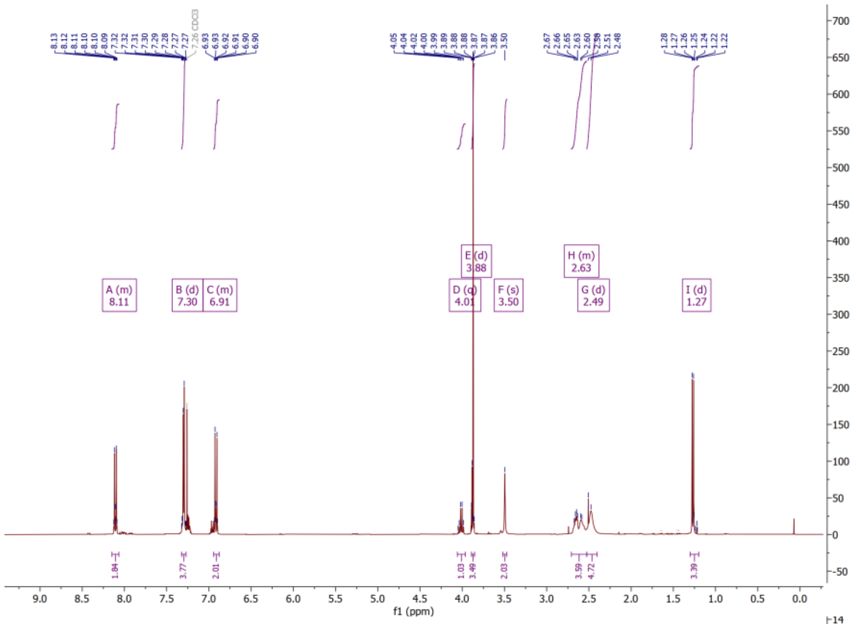

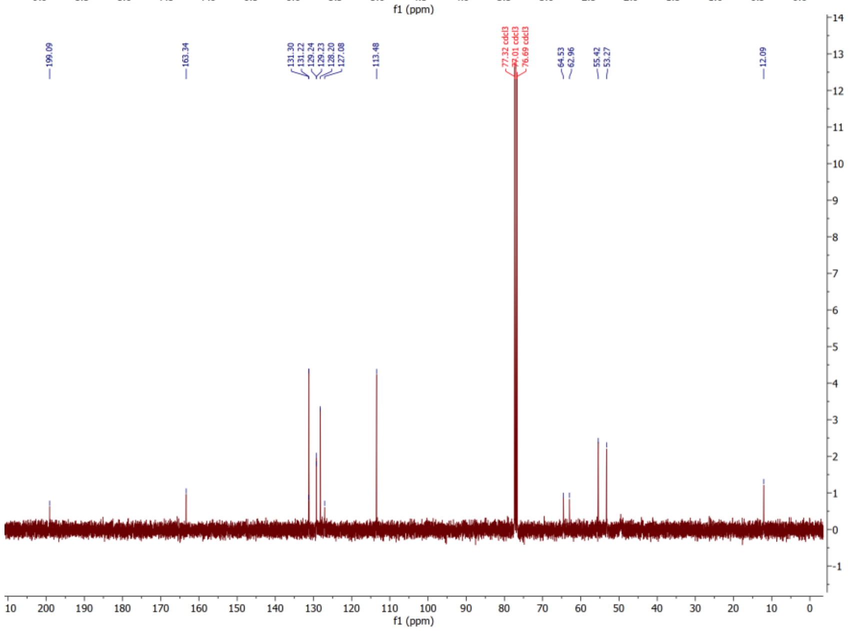

^{1}H-NMR and ^{13}C-NMR spectra for compound **5i** ($CDCl_3$)

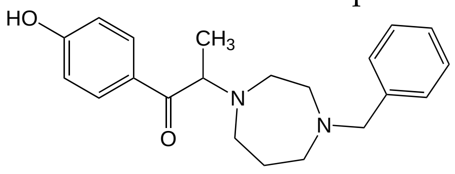

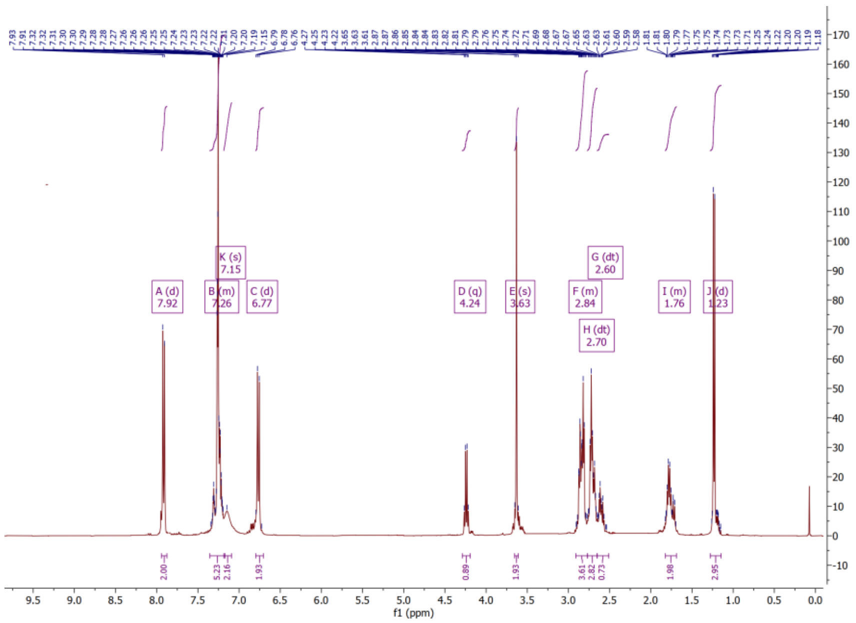

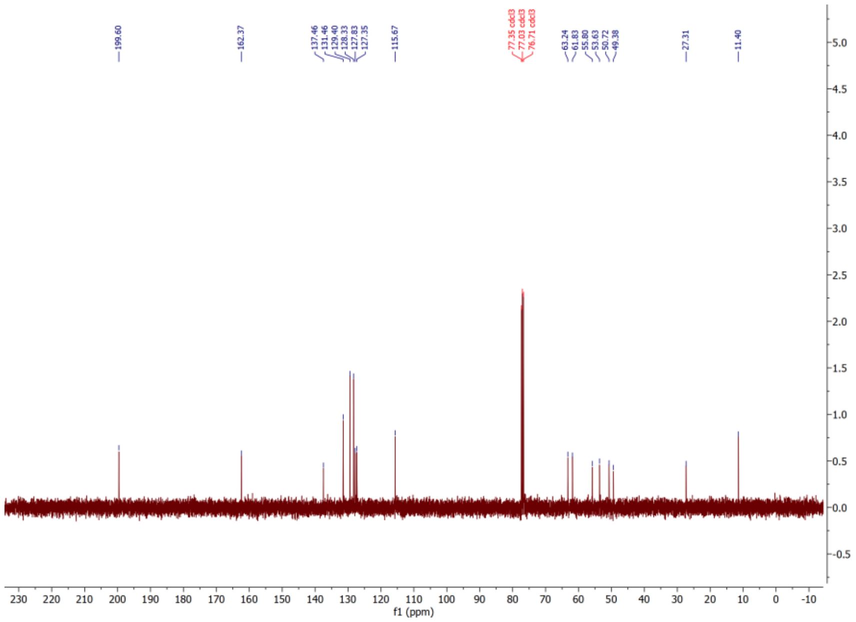

^{1}H-NMR and ^{13}C-NMR spectra for compound **5j** ($CDCl_3$)

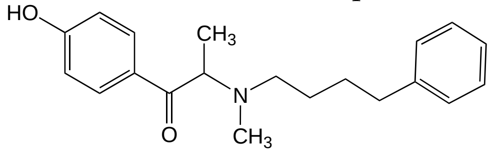

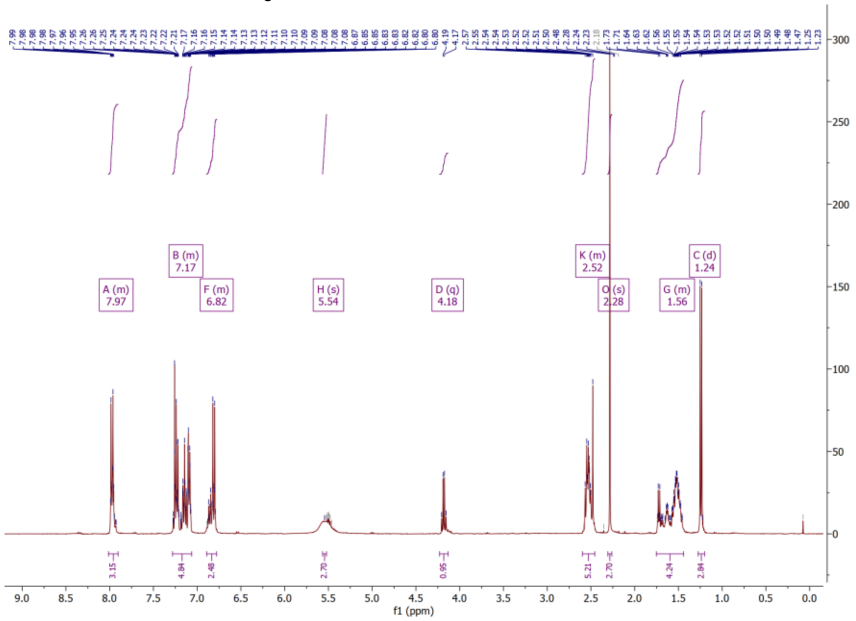

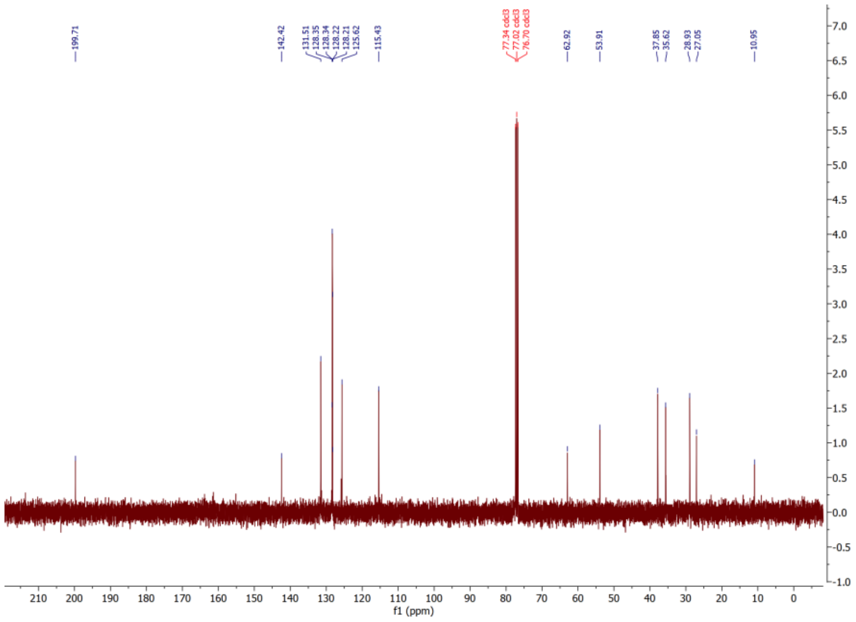

^{1}H-NMR and ^{13}C-NMR spectra for compound **5k** ($CDCl_3$)

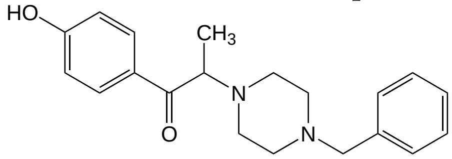

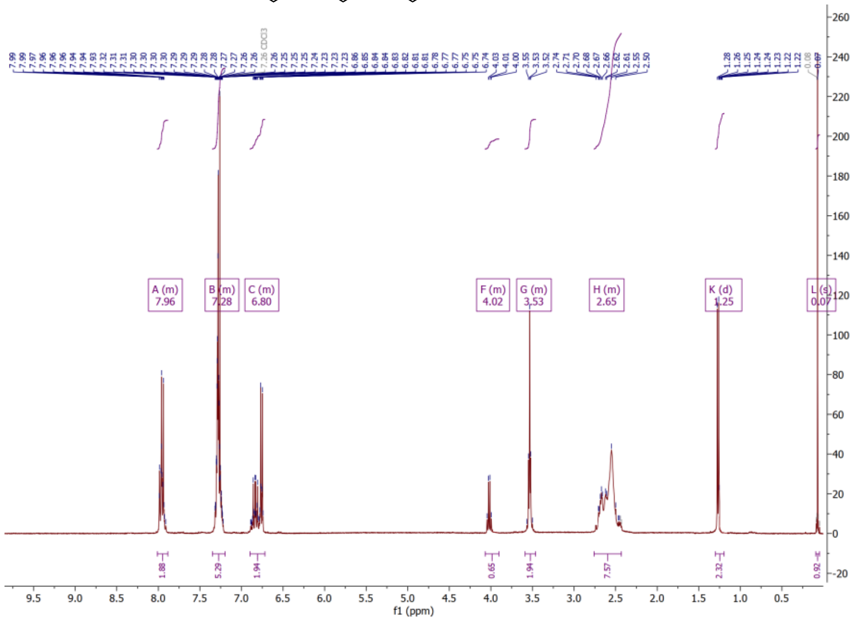

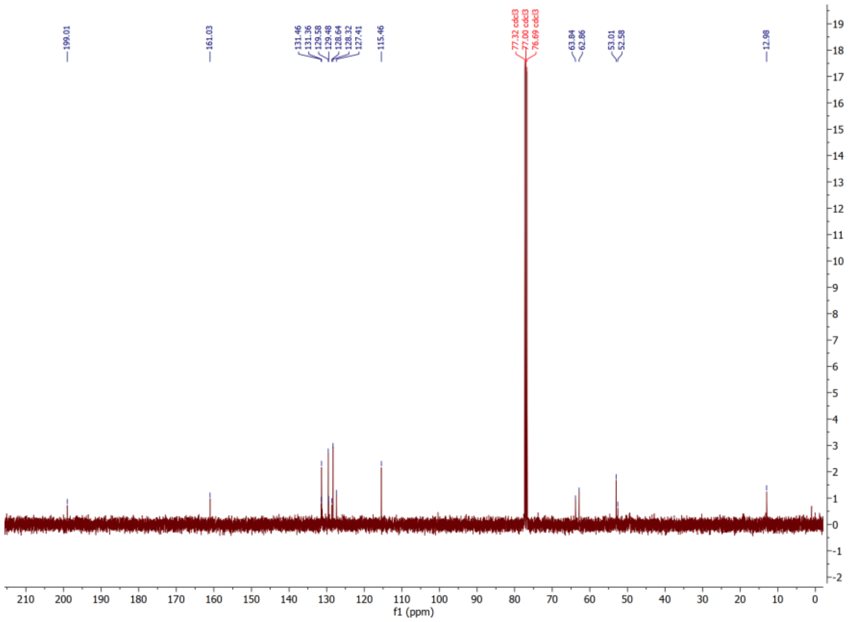

^{1}H-NMR and ^{13}C-NMR spectra for compound **5l** ($CDCl_3$)

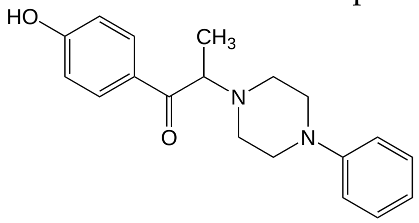

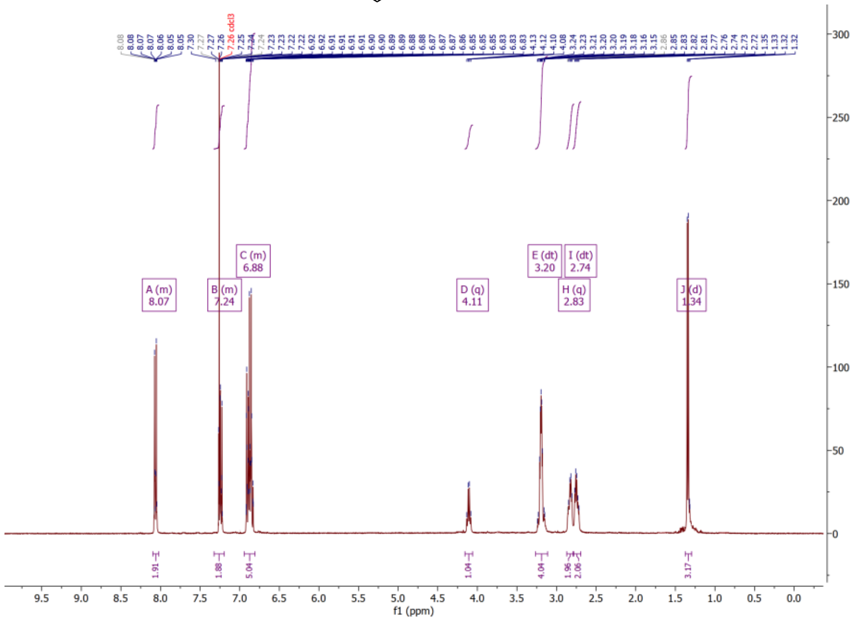

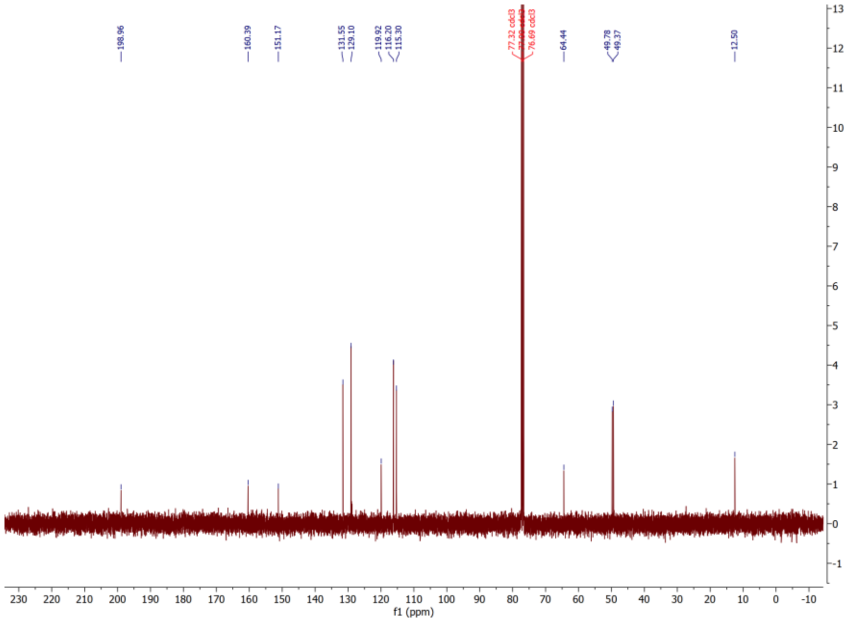

^{1}H-NMR and ^{13}C-NMR spectra for compound **5m** ($CDCl_3$)

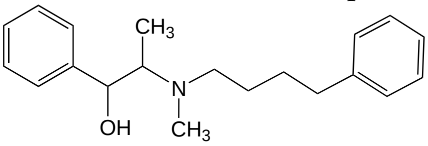

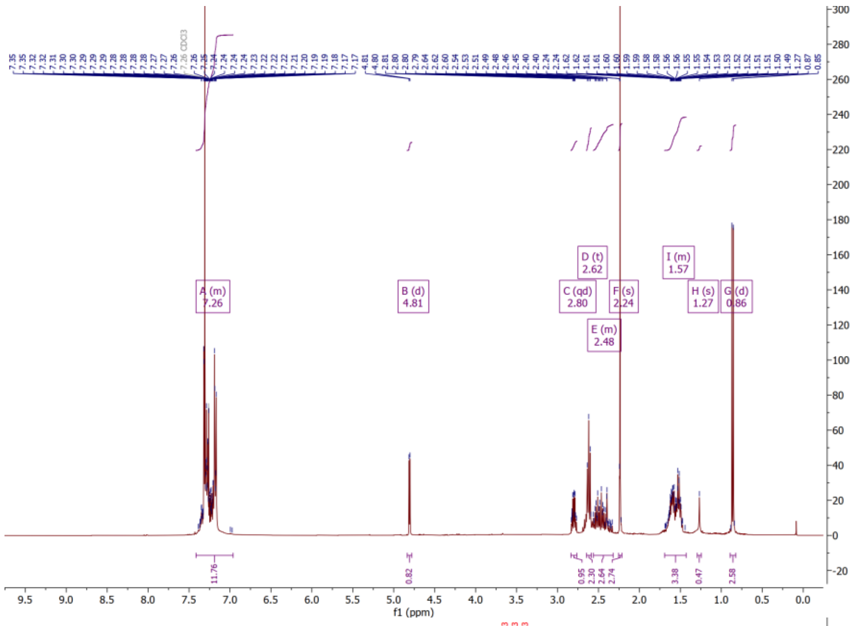

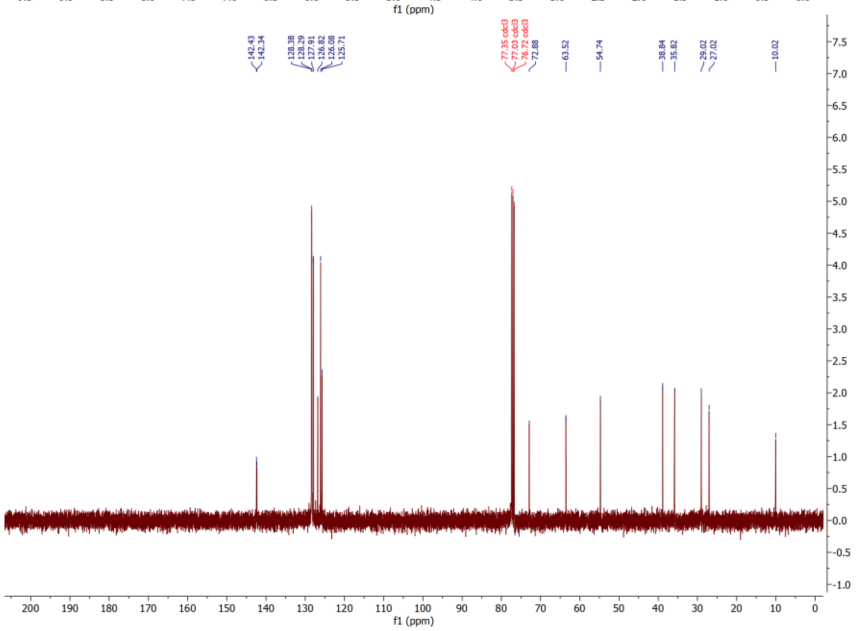

^{1}H-NMR and ^{13}C-NMR spectra for compound **5n** ($CDCl_3$)

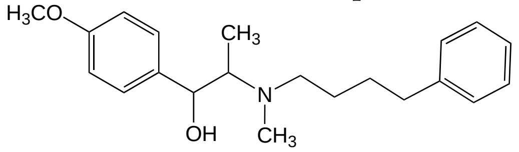

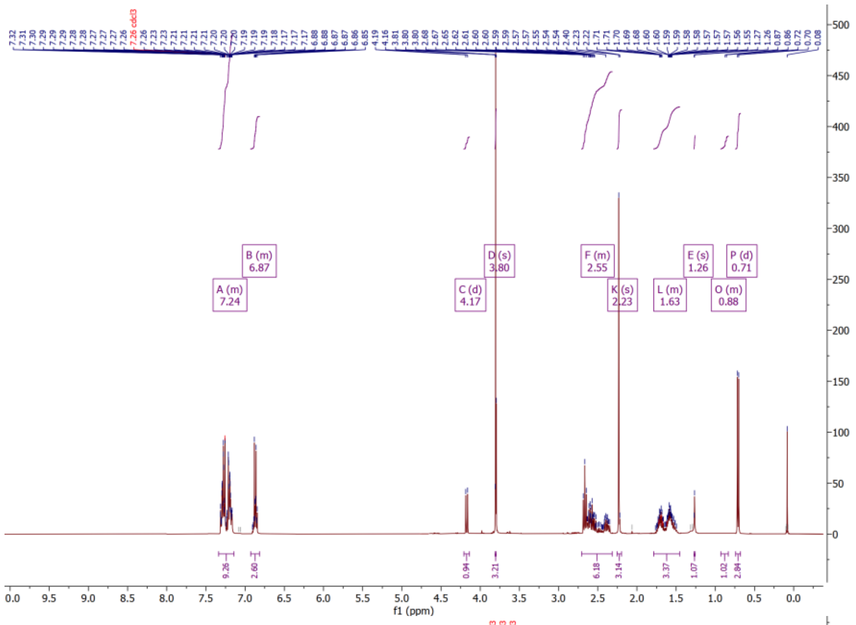

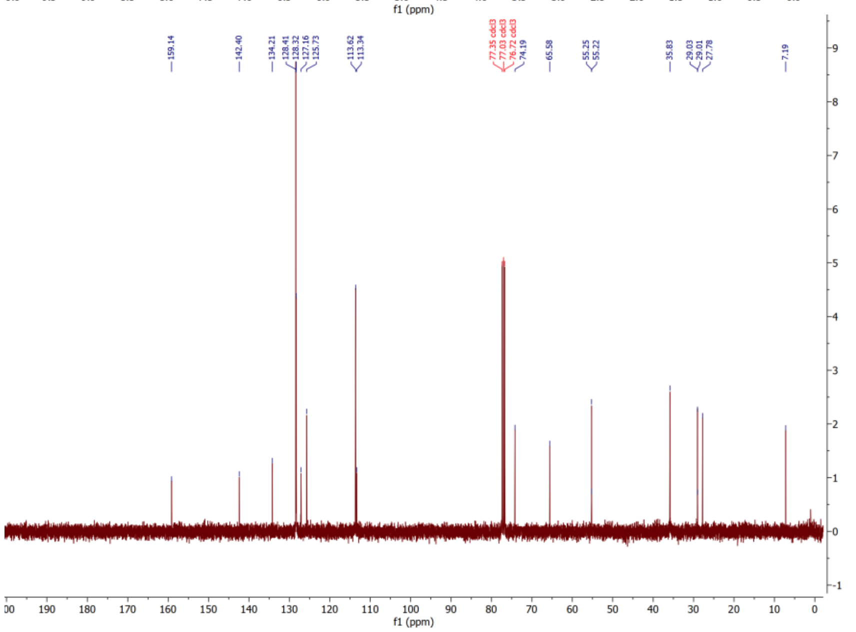

^{1}H-NMR and ^{13}C-NMR spectra for compound **5o** ($CDCl_3$)

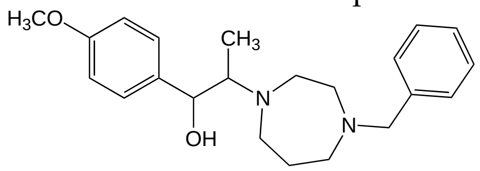

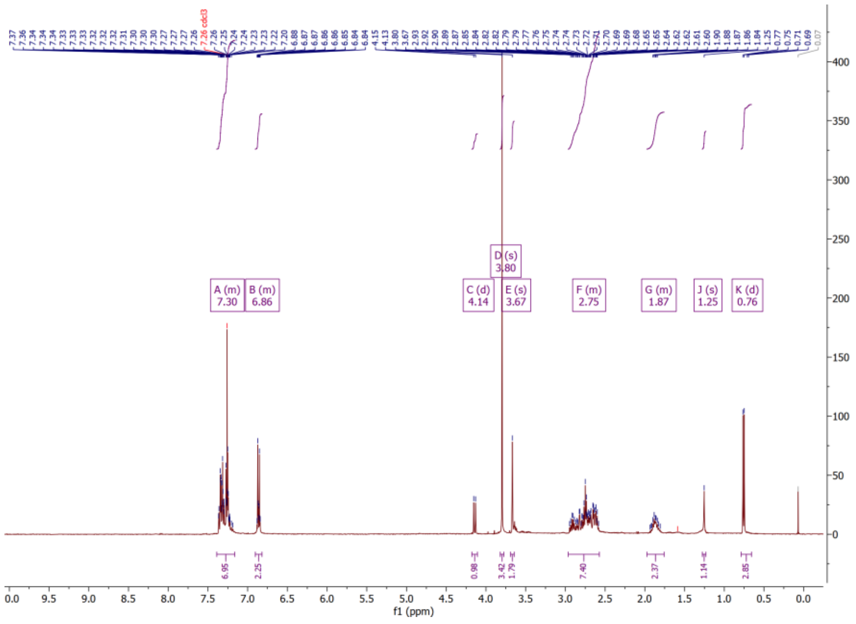

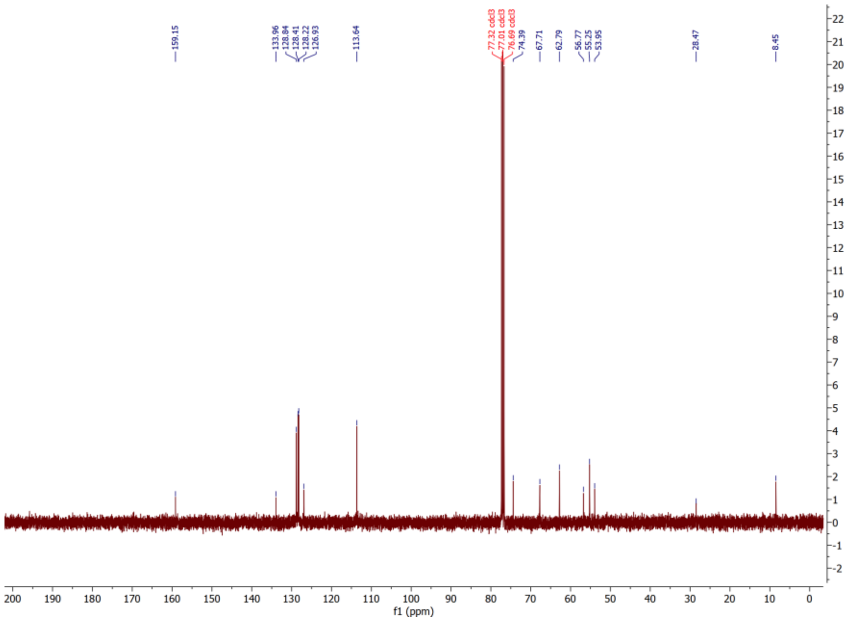

Figure S1. Competition curves for compounds **5e, 5i, 5d, 5o, 5h** and **5k** in comparison with Haloperidol, NE100 and Siramesine.

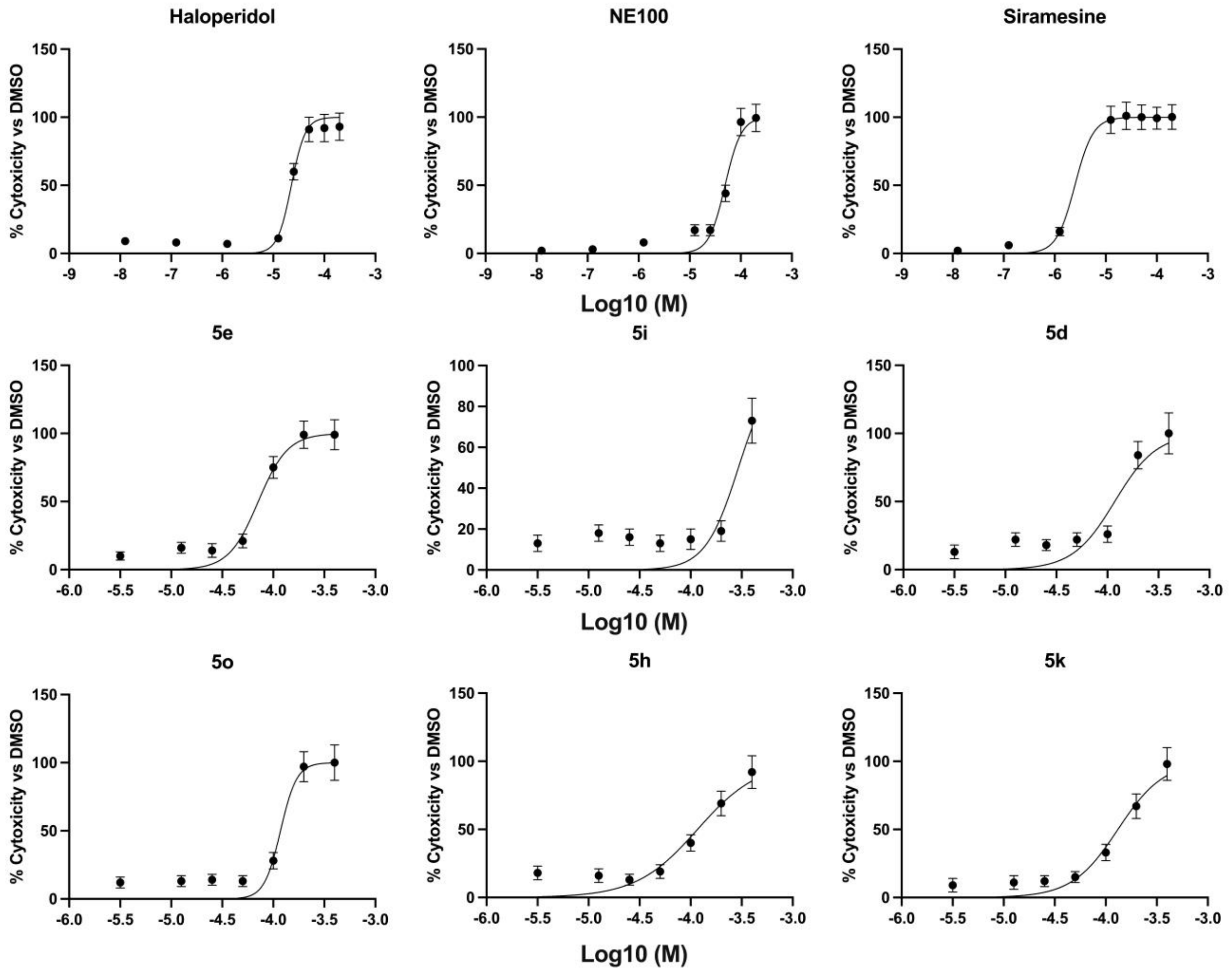

Supplement: Supplementary file 1 [file molecules-28-03431-s001.zip › molecules-2321343-supplementary.pdf]
